# Discriminative Dictionary Learning for Autism Spectrum Disorder Identification

**DOI:** 10.3389/fncom.2021.662401

**Published:** 2021-11-08

**Authors:** Wenbo Liu, Ming Li, Xiaobing Zou, Bhiksha Raj

**Affiliations:** ^1^Department of Electrical and Computer Engineering, Carnegie Mellon University, Pittsburgh, PA, United States; ^2^School of Electronics and Information Technology, Sun Yat-sen University, Guangzhou, China; ^3^Data Science Research Center, Duke Kunshan University, Suzhou, China; ^4^School of Computer Science, Wuhan University, Wuhan, China; ^5^The Third Affiliated Hospital, Sun Yat-sen University, Guangzhou, China; ^6^Language Technologies Institute, Carnegie Mellon University, Pittsburgh, PA, United States

**Keywords:** discriminative dictionary learning, autism spectrum disorder, mode seeking, machine learning, eye gaze

## Abstract

Autism Spectrum Disorder (ASD) is a group of lifelong neurodevelopmental disorders with complicated causes. A key symptom of ASD patients is their impaired interpersonal communication ability. Recent study shows that face scanning patterns of individuals with ASD are often different from those of typical developing (TD) ones. Such abnormality motivates us to study the feasibility of identifying ASD children based on their face scanning patterns with machine learning methods. In this paper, we consider using the bag-of-words (BoW) model to encode the face scanning patterns, and propose a novel dictionary learning method based on dual mode seeking for better BoW representation. Unlike k-means which is broadly used in conventional BoW models to learn dictionaries, the proposed method captures discriminative information by finding atoms which maximizes both the purity and coverage of belonging samples within one class. Compared to the rich literature of ASD studies from psychology and neural science, our work marks one of the relatively few attempts to directly identify high-functioning ASD children with machine learning methods. Experiments demonstrate the superior performance of our method with considerable gain over several baselines. Although the proposed work is yet too preliminary to directly replace existing autism diagnostic observation schedules in the clinical practice, it shed light on future applications of machine learning methods in early screening of ASD.

## 1. Introduction

Autism spectrum disorder (ASD) refers to a group of developmental disorders, including a wide range of symptoms, skills, and levels of disability. Children with ASD often suffer certain lifelong disabilities which have considerable impacts to their families (Amaral et al., 2008; Lobar, 2016). While the number of ASD children has risen dramatically in recent years, traditional ASD diagnostic approaches are both time and labor consuming, causing hinderance to early diagnosis and intervention (Zheng et al., 2013). Currently, the widely used assessments include the Autism Diagnostic Observation Schedule-Generic (ADOS-G) (Lord et al., 2000) and its revised version ADOS-2 (Gotham et al., 2007). These diagnostic methods were carefully designed to measure certain behaviors and impairments. Despite their high validity, the accompany and administration of clinically trained professionals are often required. The human-in-loop nature of these tests not only lead to time cost, but also the demand of well controlled protocols and experienced professionals.

Recent behavioral studies found that ASD individuals show abnormal scanning patterns when looking at faces (Yi et al., 2014, 2016). Similar atypical visual attention is also observed natural static images with general objects (Jones and Klin, 2013; Wang et al., 2014, 2015). In these studies, eye gaze captured by eye tracking techniques played a central role in analyzing the ASD behaviors. In the studies, a set of images are displayed on the screen and an eye tracker returns a set of the viewer's eye gaze location (x-y coordinates) on each image. The underlying motivation is that eye gaze patterns, such as the content of viewed objects, fixation durations, viewing frequency of different areas, speed/direction of saccades as well as temporal relations, may encode rich amount of ASD related information. The above studies also motivated recent attempts that use machine learning to identify ASD through abnormal visual attentions (Liu et al., 2015, 2016). These two works present early attempts to apply machine learning frameworks to identify children ASD by analyzing the eye movement patterns. In particular, the experiments are conducted on the dataset from Yi et al. (2016) where each participant is shown multiple faces, and therefore recorded with multiple face scanning sequences. Inspired by the area of interest (AOI) approach widely used in human behavior analysis, the authors consider a bag-of-words modeling where they use k-means to find areas with high fixation concentration in a data-driven manner, and encode each eye movement sequence into a single feature vector. These areas are referred to as “dictionary words,” and each feature vector is a normalized histogram representing the frequency of the eye fixations falling into different areas. Finally, kernel support vector machine (SVM) classifiers are trained and evaluated in a “leave-one-out” cross-validation manner, where each time the features of the eye movement sequences from a single participant are held for testing while the rest ones are used for training. Such framework proved to deliver promising results, which demonstrate the feasibility of using machine learning to identify ASD based on face scanning patterns.

**Figure 1:**
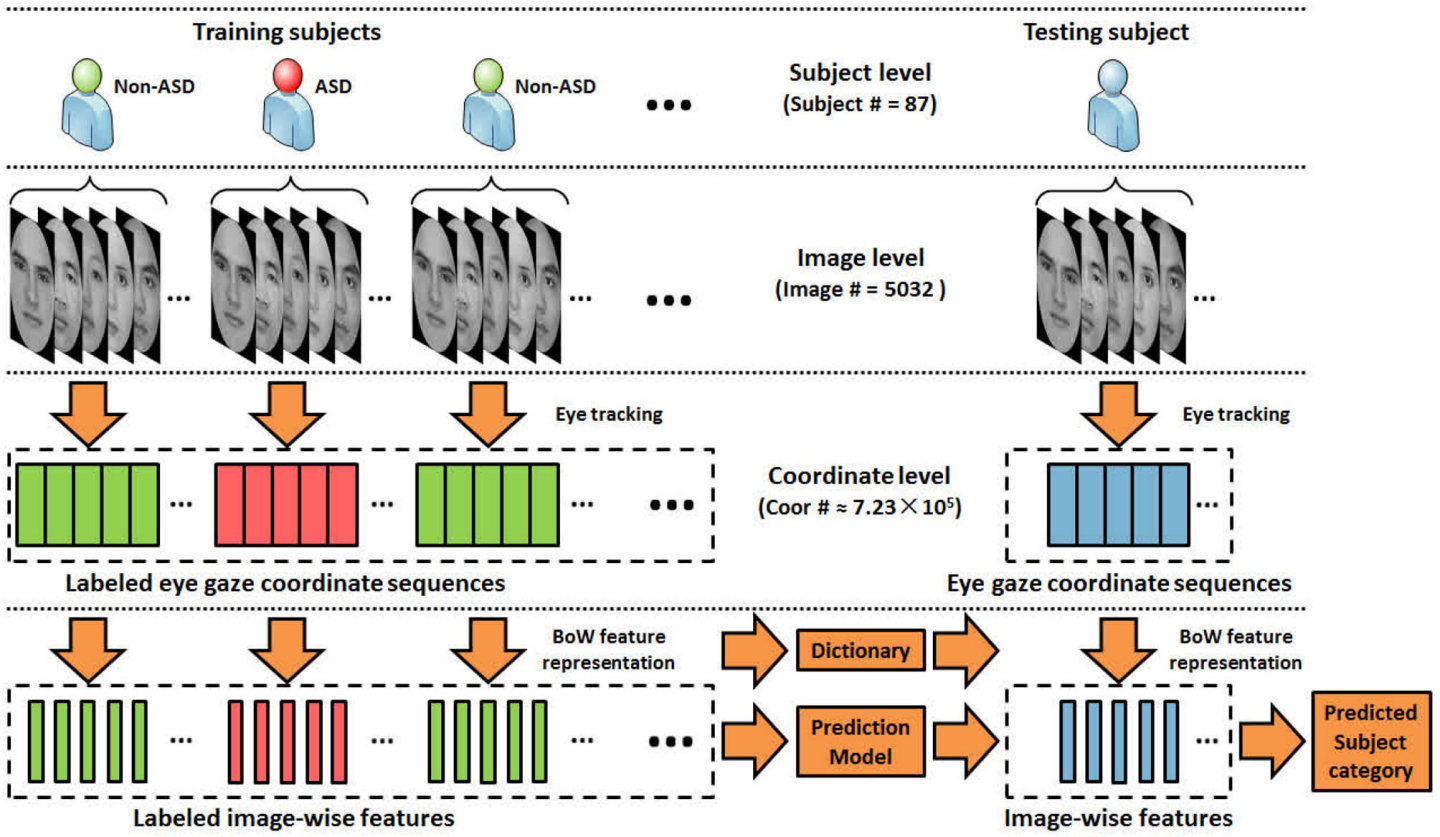
An overview of the evaluation protocol. Each subject views a set of face images, while the set of eye gaze coordinates on each viewed image are recorded using eye tracking devices. The proposed method encodes the eye gazes at image level with the BoW model.

**Figure 2:**
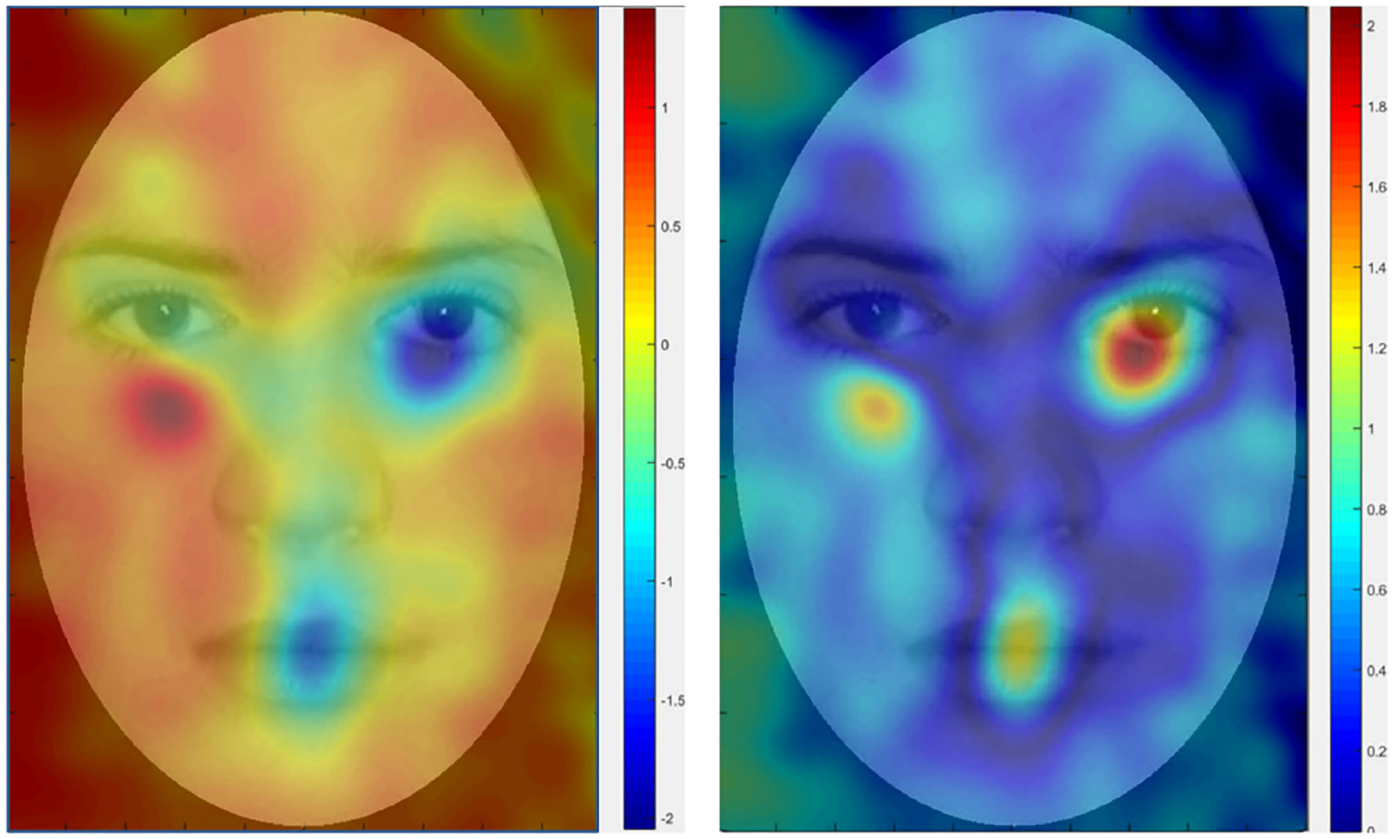
Heat maps of quality objective values at different face locations. The values encode the different visual preferences between ASD and TD subjects. TD subjects tend to fixate gazes near right eye and mouth, while ASD subjects show certain level of avoidance of eye contact. Our target is to locate these highly different regions, treating them as BoW dictionary to encode the gazes. **(Left)** Quality objective value. **(Right)** Taking absolute of the left image.

In this paper, we aim to propose improved machine learning approaches to better encode abnormal eye movement patterns and identify ASD. Our work follows the same framework and protocol proposed by Liu et al. (2016), where the gaze coordinates of diagnosed (ASD/non-ASD) subjects (Yi et al., 2015) are encoded into features under the BoW model, and classified by kernel SVMs under the “leave-one-out” cross-validation evaluation protocol, as shown in Figure 1. A major novelty of this paper lies in proposing a new dictionary learning method where high quality dictionary words are discriminatively mined. Instead of using k-means to learn words with highly concentrated attentions, our method seeks to locate words that favors concentration difference between ASD and TD gazes. We also propose a theoretically unified view toward modeling word quality, by considering **purity** and **coverage** which correspond to inter-class difference and word frequency. We model the quality objective as a product of purity and coverage, and show that such objective can be naturally approximated via kernel density estimation and optimized via dual mode seeking. Figure 2 visualizes the estimated objective values at different locations. Our contributions can be summarized as follows: (1) Our work presents an improved data-driven representation framework based on Liu et al. (2016) with strong motivations in machine learning. (2) Our work also presents an interpretable model that is well-founded in the psychology and autism research communities by showing strong connections to the well-known iMap approach (Caldara and Miellet, 2011). (3) The proposed framework leads to so far the state-of-the-art performance on two major ASD identification datasets. We believe the research conveys good contributions by benefiting a variety of downstream autism research.

## 2. Related Work

Our work is related to or partly inspired by a wide variety of previous work, ranging from psychology, psychiatry, behavior analysis to machine learning. Below we give a brief summarization of these work.

### 2.1. Psychology, Psychiatry, and Behavior Analysis in Autism Research

**Figure 3:**
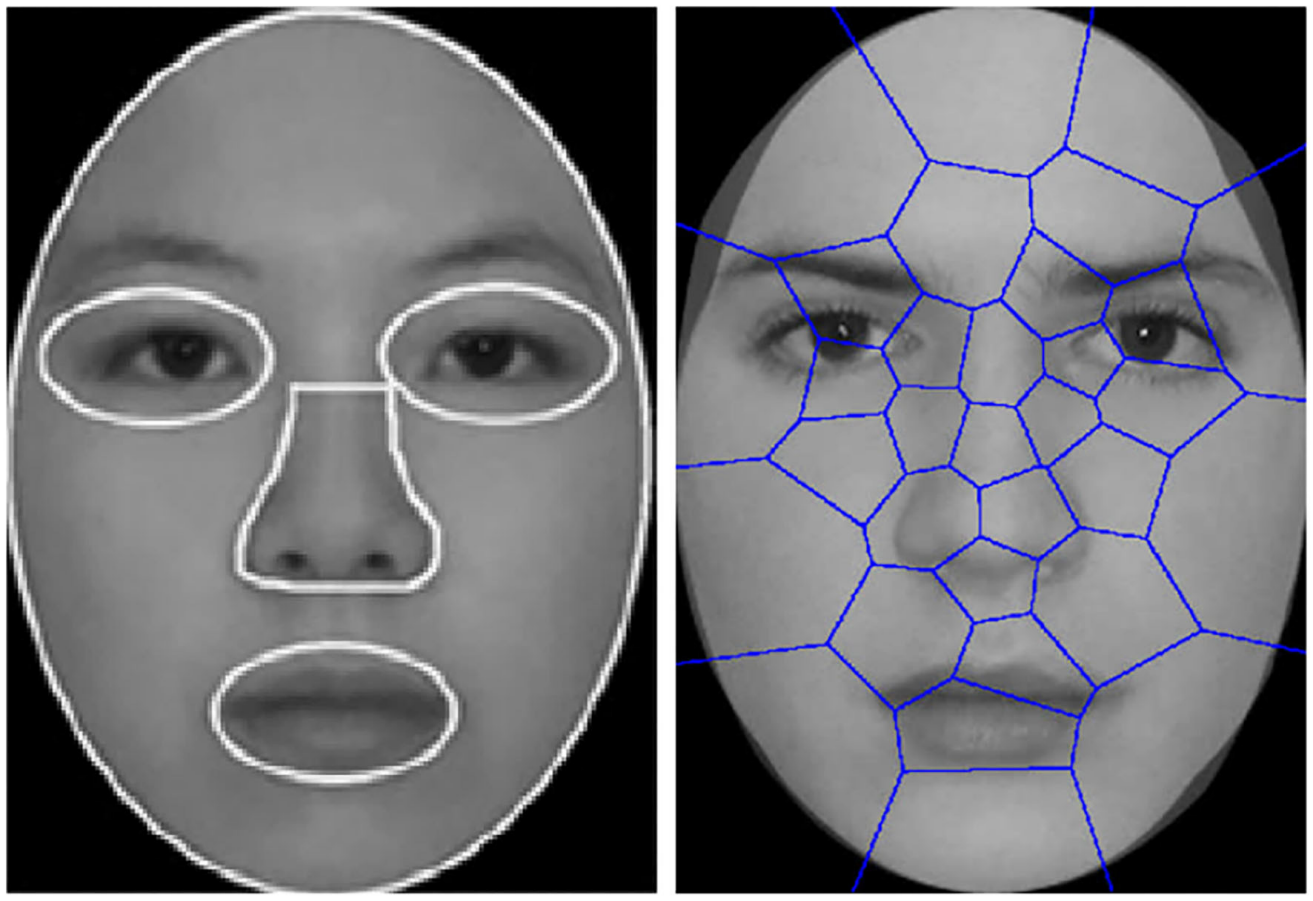
Illustration of the dictionary words projected onto the viewed image from Liu et al. (2016). **(Left)** Partitioned regions as dictionary words in AOI. **(Right)** Regions learned by k-means.

Analysis of visual attention of ASD children is theoretically supported by considerable amounts of work from the communities of psychology, psychiatry, and behavior analysis. One of the most important work is the area of interest (AOI) (Klin et al., 2002; Van der Geest et al., 2002) approach. Specifically, subjects are shown with human face images on the screen and their eye movement patterns are captured. In the data analysis step, the viewed images are manually partitioned into semantically meaningful regions (eye, nose, and mouth, etc.), with the frequency (counts) of eye fixations falling into each region counted and analyzed. A brief illustration of the AOI approach from Yi et al. (2014) is illustrated in Figure 3. Note that the regions in AOI are partitioned empirically and can be influenced by the semantic meanings. In addition, the spirit of AOI turns out to be highly related to the well-known BoW model in machine learning because counting the frequency is essentially feature encoding with histogram, whereas the partitioned regions correspond to the concept of dictionary words (or codebook) in BoW. Therefore, feature representation with BoW consists of two steps: (1) Partitioning the face image into regions that are found by data-driven approaches like k-means; (2) Counting the histogram of fixations falling into different regions and treating it as the feature This histogram is named as BoW feature representation.

Besides AOI, another method toward analyzing visual attentions is the iMap approach (Caldara and Miellet, 2011), where a heat map of visual attention is generated by smoothed eye fixations. This heat map indicate the density of eye-gaze coordinates. Different constitutes of subject would have a difference heat map. Recently, a variety of studies have reported the application of AOI and iMap in analyzing abnormal attention in ASD (Young et al., 2009; Jones and Klin, 2013; Yi et al., 2015, 2016). Again, the concept of Gaussian smoothing in iMap coincides with kernel density estimation (Rosenblatt, 1956; Parzen, 1962), and the concept of taking a difference map shows a very deep connection to our proposed discriminative framework with density difference and mode seeking (Cheng, 1995; Comaniciu and Meer, 2002). This presents a strong scientific justification of our approach from the perspective of behavior/psychology study.

### 2.2. Machine Learning for Autism Research

While the above studies form the theoretical foundations of this research, most of them are restricted to statistical studies to discover patterns indicating ASD symptoms. With the fast development and success, machine learning have been introduced to identify ASD. Using machine learning benefits the identification process from two aspects: (1) Replacing human-in-loop operations with machine learning methods makes the identification process much more scalable (Crippa et al., 2015). (2) Learning based methods can generate useful mid-level scores which reduce subjectivity in ASD identification and help better ASD diagnosis (Stahl et al., 2012; Zhou et al., 2014; Crippa et al., 2015; Wang et al., 2017).

Motivated by the abnormal eye movement patterns observed in Yi et al. (2014, 2016), Liu et al. (2015, 2016) followed the same dataset introduced in Yi et al. (2014, 2016) and proposed a machine learning based ASD identification framework using BoW representation and kernel SVM. Inspired by AOI, Liu et al. (2015, 2016) proposed to adopt data-driven approach (k-means) to group fixation coordinates into partitioned regions, and counts the frequency of fixations falling into each region. The histogram of the fixations is then used to represent the feature of the eye movement sequence. The difference between AOI and k-means is that the latter is data-driven which does not require manual partitions (see the right image in Figure 3). However, k-means tends to favor dictionary words with high sample densities but does not explicitly include discriminative class information, whereas this work aims to the issue and introduce discriminative class information into dictionary learning. Besides Liu et al. (2015, 2016), another closely related work is Jiang and Zhao (2017) where deep network is used to identify ASD by analyzing the viewed contents of test subjects.

### 2.3. General Machine Learning Research

The concept of adding supervised discriminative information to dictionary learning is not new. A number of previous work on sparse dictionary learning have reported improvements over unsupervised dictionary learning when label information is incorporated (Mairal et al., 2008; Jiang et al., 2011; Yang et al., 2011). While these work presented elegant optimization frameworks for learning sparse dictionaries, their method can not be directly applied to our task given the low dimensional input.^1^ Of course, one may argue that higher dimensional inputs can be obtained by sampling the time series of the coordinates in a relatively long window. However, this is much less supported by previous clinical studies in psychology and behavior analysis, and often does not work well since high-order temporal context information along temporal dimension are usually quite random and fixational eye movements have large effects on visual perception than saccades (Krauzlis et al., 2017).

The discrete and non-convex nature of k-means makes it even more difficult to incorporate discriminative label information to the learning process similar to sparse dictionary learning. A typical method to heuristically incorporate discriminative information is to learn class-specific dictionary by performing k-means on the class-wise data subsets (Altintakan and Yazici, 2015). Other related methods include the descriptive word ranking (Zhang et al., 2009) as well as large-margin clustering using SVM and iterative cross-validation (Doersch et al., 2012; Singh et al., 2012).

One related work which partly inspired our proposed framework is Doersch et al. (2013), where the authors discover mid-level image patches with discriminative mode seeking, and formulate the mode seeking process as a constrained optimization problem. While resembling their method in high-level motivations, our work differs from them in several aspects: (1) Doersch et al. (2013) is heavily tailored to handle image classification problems and very high dimensional features. While our method addresses a completely different application. (2) We observe that directly using density ratio as in Doersch et al. (2013) sometimes leads to super large values and undesired learning behaviors when the denominator is small. Instead we consider an alternative purity measurement term where the purity is normalized between 0 and 1. (3) Our model considers the joint optimization of purity and coverage, while the density ratio somewhat discards the coverage information of dictionary. (4) We show that the proposed optimization framework can be elegantly formulated as a supervised mean shift, which is considerably simpler than the constrained optimization in Doersch et al. (2013).

## 3. Learning Discriminative Bag-of-Words Dictionary

Dictionary learning presents an important problem in BoW representation as the quality of learned dictionary words has direct impacts on the quality of represented features. Often, one would hope that the dictionary can encode as much discriminative information as possible, such that the feature coefficients on this dictionary show significant inter-class differences which benefit the classification task. An important question one may ask is: How to quantitatively measure the quality of a given dictionary?

### 3.1. Notations and Definitions

Before delving into technical details, we list the notations and their definitions in this subsection for the algorithmic clarity. For the rest of the paper, we use $X=\left\{x_{i}|x_{i}\in {\mathbb{R}}^{2},i\in 1,\ldots ,N\right\}$ to denote the entire set of 2D eye fixation coordinates on all the viewed faces from all the participants in the training set, where *N* is the total number of coordinate samples. We use $X^{+}=\left\{x_{i}|i\in 1,\ldots ,N^{+}\right\}$ to denote the set of coordinates from the participants diagnosed with ASD in the training set, and similarly $X^{-}=\left\{x_{i}|i\in 1,\ldots ,N^{-}\right\}$ the set of coordinates from the rest participants, where ***X*** = ***X***^+^ ∪ ***X***^−^.

Toward encoding the fixation coordinates with BoW representation, we assume that the coordinates are grouped into *K* clusters with a cluster partitioning ***C*** ∈ {1, …, *K*}^*N*^, where ***C*** is a labeling configuration of cluster ids for each coordinate. Each cluster corresponds to a dictionary word. Our goal is to find an optimized cluster partitioning that leads to improved dictionary quality and BoW representation.

### 3.2. A Unified View Toward Dictionary Quality

In Doersch et al. (2013), the authors proposed the concept of **purity** to measure how discriminative a dictionary word is, and **coverage** to measure how representative it is. We follow this idea to learn dictionaries that have larger values in both terms. Given a certain cluster partition ***C*** and the cluster index *k*, the purity for positive class *P*^+^(*k* ∣ ***C***) can be modeled as:

(1) $$\begin{array}{r} P^{+}\left(k|C\right)=\frac{N^{+}\left(k|C\right)}{N^{+}\left(k|C\right)+N^{-}\left(k|C\right)}, \end{array}$$

where *N*^+^(*k* ∣ ***C***) and *N*^−^(*k* ∣ ***C***), respectively, denotes the numbers of positive and negative samples assigned to cluster partition C. Again, note that such measurement differs from the density ratio in Doersch et al. (2013), in the sense that Equation (1) is normalized between 0 and 1. Similarly, the purity of negative class can be defined as:

(2) $$\begin{array}{r} P^{-}\left(k|C\right)=\frac{N^{-}\left(k|C\right)}{N^{+}\left(k|C\right)+N^{-}\left(k|C\right)}. \end{array}$$

While it is desirable to increase the dictionary purities for both classes, increasing the purity of both positive and negative classes is contradicting in the same word. What truly matters is the difference of sample numbers and its ratio vs. the word size. As a result, we look into the following purity measure:

(3) $$\begin{array}{r} P\left(k|C\right)=\frac{\left|N^{+}\left(k|C\right)-N^{-}\left(k|C\right)\right|}{N^{+}\left(k|C\right)+N^{-}\left(k|C\right)}, \end{array}$$

which is able to measure the level of purity for both classes with a unified representation. On the other hand, the coverage for positive and negative class can be modeled as:

(4) $$\begin{array}{r} C\left(k|C\right)=N^{+}\left(k|C\right)+N^{-}\left(k|C\right). \end{array}$$

A dictionary ideally should have good purity and coverage simultaneously. A natural way is to treat the product of both benchmarks as the objective, which shares similar spirit to the f-measure.^2^ Therefore, the word quality can be estimated as:

(5) $$\begin{array}{r} Q\left(k,C\right)≜P\left(k|C\right)C\left(k|C\right)=|N^{+}\left(k|C\right)-N^{-}\left(k|C\right)| \end{array}$$

The problem of finding a good dictionary word can therefore be formulated as maximizing the quality estimation objective with respect to *k* and ***C***:

(6) $$\begin{array}{r} \underset{C}{\max}\underset{k}{\sum }Q\left(k,C\right) \end{array}$$

### 3.3. Approximating With Kernel Density Estimation

Directly optimizing the objective in Equation (6) is difficult since the optimization is non-continuous, non-convex, and the solution space of ***C*** is huge. Our approach here is to approximate with kernel density estimation and mode-seeking. Specifically, when the size of each dictionary word is reasonably small, a good approximation to *N*^+^(*k* ∣ ***C***) and *N*^−^(*k* ∣ ***C***) is the local density estimator:

(7) $$\begin{array}{r} \hat{P}\left(x_{k}|X^{+}\right)\propto N^{+}\left(k|C\right)\;\hat{P}\left(x_{k}|X^{-}\right)\propto N^{-}\left(k|C\right) \end{array}$$

where ***x***_*k*_ is the location of the *k*-th dictionary word in feature space. In addition, we define $\hat{P}\left(x_{k}|X^{+}\right)$ to be the following Gaussian kernel density estimator:

(8) $$\begin{array}{rc} \hat{P}\left(x|X^{+}\right) & ≜\frac{c_{d}}{Nh^{d}}\underset{x_{i}\in X^{+}}{\sum }\exp\left(-\frac{||x-x_{i}|{|}^{2}}{2h^{2}}\right)\\ \hat{P}\left(x|X^{-}\right) & ≜\frac{c_{d}}{Nh^{d}}\underset{x_{i}\in X^{-}}{\sum }\exp\left(-\frac{||x-x_{i}|{|}^{2}}{2h^{2}}\right) \end{array}$$

where *d* = 2 is the dimension, *h* is the bandwidth that controls the kernel smoothness, and $c_{d}=2\pi^{\left(-d/2\right)}$ is a normalization constant. The word quality located at ***x*** can thus be estimated as:

(9) $$\begin{array}{r} \begin{array}{c} Q\left(x\right)=|\hat{P}\left(x|X^{+}\right)-\hat{P}\left(x|X^{-}\right)| \end{array} \end{array}$$

### 3.4. Finding *Q*(*x*) Local Maxima With Dual Mode Seeking

Our goal is to find a set of local maxima of *Q*(*x*) which indicate the locations of high quality words. Note that Equation (9) is a continuous function with respect to ***x***. This allows one to optimize it with respect to ***x*** using gradient ascent. Since Equation (9) contains absolute values, we consider the alternative objective:

(10) $$\begin{array}{r} Q^{*}\left(x\right)=\hat{P}\left(x|X^{+}\right)-\hat{P}\left(x|X^{-}\right) \end{array}$$

Assuming that the gradient ascent/descent process guarantees the monotonic increasing/decreasing of Q^*^(***x***), we have the following theorems:

**Proposition 1:** *Q*(***x***) = −Q^*^(***x***), ∀***x*** ∈ {***x***|Q^*^(***x***) < 0}.

**Remark:** The proof is omitted as it is strightforward. Proposition 1 indicates that the landscape of *Q*(***x***) is equal to flipping the negative part of Q^*^(***x***) as positive.

**Proposition 2:** Gradient ascent on *Q*(***x***) is equal to gradient ascent on Q^*^(***x***), ∀***x*** ∈ {***x***|Q^*^(***x***) > 0}.

**Proposition 3:** Gradient ascent on *Q*(***x***) is equal to gradient descent on Q^*^(***x***), ∀***x*** ∈ {***x***|Q^*^(***x***) < 0}.

**Remark:** Proposition 2 and 3 can be directly concluded from Proposition 1. As a result, performing mode seeking on *Q*(***x***) can be alternatively done by performing dual gradient ascent/descent on Q^*^(***x***) with respect to the gradient ∇Q^*^(***x***). To simplify the computation, note that we have:

(11) $$\begin{array}{r} \nabla \hat{P}\left(x|X^{+}\right)=\frac{1}{h^{2}}\hat{P}\left(x|X^{+}\right)\left(x_{m^{+}}-x\right)\\ \nabla \hat{P}\left(x|X^{-}\right)=\frac{1}{h^{2}}\hat{P}\left(x|X^{-}\right)\left(x_{m^{-}}-x\right) \end{array}$$

where $x_{m^{+}}$ is the weighted mean of positive data samples weighted by kernels:

(12) $$\begin{array}{r} x_{m^{+}}=\frac{\sum_{x\in X^{+}}\exp\left(-||x-x_{i}|{|}^{2}/2h^{2}\right)x_{i}}{\sum_{x\in X^{+}}\exp\left(-||x-x_{i}|{|}^{2}/2h^{2}\right)} \end{array}$$

$x_{m^{-}}$ is defined similarly. The gradient of objective function is therefore computed as:

(13) $$\begin{array}{r} \nabla Q^{*}\left(x\right)=\frac{1}{h^{2}}\left[\hat{P}\left(x|X^{+}\right)\left(x_{m^{+}}-x\right)-\hat{P}\left(x|X^{-}\right)\left(x_{m^{-}}-x\right)\right] \end{array}$$

One could see that Equation (13) is basically a weighted combination of the mean shift vectors (Comaniciu and Meer, 2002) from positive and negative samples, where the weights are the kernel densities. Accordingly, one may consider the following dual mode seeking step to find local maxima of *Q*(***x***) (see **Algorithm 1**):

**Algorithm 1:** Dual mode seeking.

1: Estimate word quality located at location ***x***: $\hat{P}\left(x_{i}|X^{+}\right)-\hat{P}\left(x_{i}|X^{-}\right)$
2: **while** not converged **do**
3: **if** $\hat{P}\left(x_{i}|X^{+}\right)-\hat{P}\left(x_{i}|X^{-}\right)>0$ **then**
4: perform mode seeking with $\nabla Q^{*}\left(x_{i}\right)$ until convergence (gradient ascent)
5: **else if** $\hat{P}\left(x_{i}|X^{+}\right)-\hat{P}\left(x_{i}|X^{-}\right)<0$ **then**
6: perform mode seeking with $-\nabla Q^{*}\left(x_{i}\right)$ until convergence (gradient descent)
7: **end if**
8: **end while**

### 3.5. Dual Mode Seeking as Supervised Mean Shift

In reality, one does not need to explicitly flip the sign of ∇Q^*^(***x***) in order to perform dual mode seeking. Let *y*_*i*_ ∈ {1, − 1} indicates the label of ***x***_*i*_, the Equation (13) can be re-written as:

(14) $$\begin{array}{r} \nabla Q^{*}\left(x\right)=\frac{c_{d}}{Nh^{d+2}}\left[\overset{N}{\underset{i=1}{\sum }}y_{i}k\left(x,x_{i}\right)\right]\left[\frac{\sum_{i=1}^{N}y_{i}k\left(x,x_{i}\right)x_{i}}{\sum_{i=1}^{n}y_{i}k\left(x,x_{i}\right)}-x\right] \end{array}$$

where we have:

(15) $$\begin{array}{r} \overset{N}{\underset{i=1}{\sum }}y_{i}k\left(x,x_{i}\right)=\overset{N}{\underset{i=1}{\sum }}y_{i}\exp\left(-\frac{||x-x_{i}|{|}^{2}}{2h^{2}}\right)=\frac{Nh^{d}}{c_{d}}Q^{*}\left(x\right) \end{array}$$

Note that dividing Equation (14) with $\overset{n}{\underset{i=1}{\sum }}y_{i}k\left(x,x_{i}\right)$ actually gives a generalized form of mean shift. Also, the sign of *g*(***x***) is exactly determined by $\overset{n}{\underset{i=1}{\sum }}y_{i}k\left(x,x_{i}\right)$. One may cancel the flipping sign of dual mode seeking simply by iteratively shifting with the following mean shift vector:

(16) $$\begin{array}{r} m\left(x\right)=\frac{\sum_{i=1}^{N}y_{i}k\left(x,x_{i}\right)x_{i}}{\sum_{i=1}^{n}y_{i}k\left(x,x_{i}\right)}-x \end{array}$$

Note that an interesting aspect of the above mode seeking algorithm (Equations 14–16) is that it can be viewed as a generalized form of supervised mean shift algorithm, where the labels *y*_*i*_ introduce class-aware discriminative information into the learning process.

### 3.6. Convergence With Back Tracking Line Search

Unfortunately, unlike the conventional mean shift, performing gradient ascent with Equation (16) does not guarantee the monotonic increase of gradient and algorithm convergence, since the sum of kernel weights contains negative terms. This often happens when the densities of positive and negative classes are approximately equal to each other. In this case the denominator of Equation (16) is very small, leading to relatively large shifting vector or potential numerical issues. This can be practically solved by adaptive step size normalization with respect to the denominator and step size reduction with back tracking line search. Whenever the quality objective value of the next step is not increased, back tracking line search multiplies the current step size with 0.5. This guarantees the monotonic increase of the objective and the algorithm convergence. In practice we observe that mean shift with Equation (16) works well at most feature space positions, and the need for performing back tracking line search is reduced very fast as the density of one class quickly dominates over another.

## 4. Summary

### 4.1. Method Overview

Zooming out a bit, we briefly recap our full picture. We started from the motivation to capture local modes that maximize the difference between ASD and non-ASD subjects on the attention maps. Our goal is to automatically identify these modes through a data-driven method in contrast to manual selection. In section 3.2, we start by defining quantitative measures of the dictionary (cluster) quality with purity and coverage. We then define the dictionary quality as the multiplication of purity and coverage. We approximate the dictionary quality with kernel density estimation in section 3.3, and further approximate the optimization of dictionary as dual mode seeking in section 3.4. Finally, we show that the proposed dual mode seeking method can be generalized into a supervised mean shift form in section 3.5, and addresses convergence issues in section 3.6.

### 4.2. From Discriminative Modes to BoW Representation

The discriminative mode seeking algorithm in section 3 returns a set of local maxima of *Q*(*x*) which indicate locations of high quality dictionary words. The subsequent question is how to transform these maxima into BoW representation by learning a particular clustering configuration ***C*** that favors these locations.

To this end, we consider a mean shift based clustering method to obtain the dictionary words and ***C***. The idea here is to initialize a set of kernel locations ***x*** with the coordinate samples and iteratively apply discriminative mode seeking in section 3 to each kernel for adequate number of iterations. This will basically shift each of the kernel from its initial feature space location to local maxima of *Q*(*x*) through gradient ascent. We then treat these shifted kernels as data samples and use k-means to obtain a total of *K* cluster centroids which are mostly located on the *Q*(*x*) maxima.

Specifically, we use all the fixation coordinate samples in the training set for density estimation. For speed purpose, we sample 1 out of 20 training coordinates to initialize the kernel locations, and perform 30 rounds of mean shifts on these kernels. We keep these settings the same across all our experiments. Once obtaining the cluster centroids, we assign each coordinate sample to the nearest centroid, therefore obtaining a cluster labeling ***C*** and the dictionary words. We then use the words to compute the BoW feature to encode the fixation coordinates for each sequence.

## 5. Experimental Results

In this section, we report comprehensive evaluations of our method on several datasets.

### 5.1. Dataset Description

We consider two datasets in this paper. The first one, child dataset (Yi et al., 2015), includes three groups of children: 29 4-to 11-year-old Chinese children with ASD, 29 Chinese TD children with matched age, and another group of 29 Chinese TD children matched with IQ. All children with ASD were diagnosed by experienced clinicians and met the diagnostic criteria for autism spectrum disorder according to the DSM-IV (American Psychiatric Association, 1980). Participants were asked to view three Chinese faces (same-race faces) and three Caucasian faces (the other-race faces), and try to memorize and recognize the faces. Note that the sensitive information including child's face, name, age were removed by the authors (Yi et al., 2016) in the dataset. More details of the participants, the material, and the experimental procedures are provided in Yi et al. (2016).

The second one, adult dataset, focuses on adolescents and young adults, and is a slightly cleaned up version of the dataset used in Yi et al. (2014) and Liu et al. (2015). The dataset includes 19 ASD and 46 non-ASD young adults. As a result, the results on the adult dataset between this paper and Liu et al. (2015) may have certain mismatches, and are not directly comparable.

For both datasets, the eye gaze movements of each person were recorded by a Tobii T60 eye tracker. A set of face images 700 ∗ 500 are displayed on the screen and eye gaze of each subject is automatically estimated, returning a series of projected coordinates.

### 5.2. Evaluation Protocol

Following Liu et al. (2015, 2016), we evaluate the proposed method by leave-one-out cross-validation testing, where each subject is consecutively held out for testing while the rest are used for training. By doing this each time we divide the image-level BoW features into two sets: one for testing and the other for training a prediction model. Following Liu et al. (2015, 2016), we train an RBF kernel SVM as the prediction model, and predict the test subject score as the mean over the soft SVM prediction scores on the images viewed by each test subject. Finally, a global threshold *T* is set for all testing subjects to obtain the subject-level predictions. For the fairness of comparison, we vary and search the hyperparameters of all comparing methods and report the best performance. Specifically, For the proposed method and baselines which include the k-means clustering step, we search the number of clusters within {35, 40, 45, 50, 55, 60, 65, 70}. We also search the γ and *C* values in kernel SVM for all comparing methods, by varying them as exponentials of 2. The search ranges of γ and *C* are set to 2^−6^ ~ 2^0^ and 2^6^ ~ 2^16^, respectively.

### 5.3. Evaluation Benchmarks

In our experiment, we consider the following benchmarks to quantitatively evaluate the prediction performance:

**Accuracy (Acc):** The number of correctly predicted subjects vs. the total number of subjects.

**Area under the curve (AUC):** The total area under the ROC curve vs. the whole area. And the ROC curve is a set of (subject-level) true positive rates vs. false positive rates obtained by synchronously varied the global threshold *T* for all testing predictions.

**Purity:** To analyze the level of determinativeness of the dictionaries learned by different methods, we also visualize the dictionary purity profile of comparing methods.

**Sensitivity:** Ratio of correct true positives vs. positives.

**Specificity:** Ratio of correct true negatives vs. negatives.

### 5.4. Baselines

We compare our method with several dictionary learning baselines that are closely related to BoW representations:

**K-means**. As described and reported in Liu et al. (2016).

**Class k-means**. K-means on both positive and negative data separately with approximately the same number of clusters.

**Mean shift**. Applying the conventional mean shift (Comaniciu and Meer, 2002) on all the data, followed by k-means dictionary learning.

**Class mean shift**. mean shift on both positive and negative data separately, followed by k-means dictionary learning.

**Disc mode seek**. Applying discriminative mode seeking (Doersch et al., 2013) on all the data, followed by k-means dictionary learning.

Note that both class k-means and class mean shift can be regarded as variants of Altintakan and Yazici (2015) where the concept of class-aware BoW representations is adopted to our problem. In addition, the bandwidths of density estimators in mean shift, class mean shift, and the proposed method are also cross-validated.

### 5.5. Main Results on Child Dataset

Following Liu et al. (2016), we comprehensively evaluate the proposed method and baselines on the complete child dataset as well as controlled scenarios where the non-ASD group is divided into IQ-matched and age-matched groups. We denote these two settings as “ASD—TD-IQ” and “ASD—TD-Age,” respectively. Results of the comparing methods are reported in Table 1, indicating that the proposed overall performs better.

**Table 1:** Results on child dataset with different TD Groups.

**Dataset**	**All data**		**ASD—TD-IQ**		**ASD—TD-Age**	
**Eval metric**	**Acc**	**AUC**	**Acc**	**AUC**	**Acc**	**AUC**
K-means	88.51	89.63	86.21	88.94	84.48	85.37
Class K-means	87.36	90.79	83.91	84.74	82.76	85.38
Mean shift	89.66	92.51	87.93	88.59	**87.93**	**88.59**
Class mean shift	88.51	92.83	86.21	89.08	86.21	86.87
Disc mode seek	89.66	92.64	87.93	89.34	**87.93**	87.03
**Proposed**	**91.95**	**93.40**	**89.66**	**90.96**	**87.93**	87.45

*Bold values indicate best performance*.

For child dataset, each child is shown with face images from two sources: Faces from the same race (Asian) and faces from other races (Caucasian). This is another typical setting in psychology study to analyze the ASD behavior. Following this setting, we subdivide our dataset into two subsets, and conduct the same evaluation. Table 2 shows the results of the proposed method and comparing baselines on the child dataset. One could see that compared with other baselines, our method has the highest accuracy (91.95%) and AUC (93.4%) on the full dataset as well as on the same race and other race subsets. This shows the benefit from the improved dictionary word quality using our method.

**Table 2:** Results on child dataset with different face subsets.

**Dataset**	**All data**		**Same race**		**Other race**	
**Eval metric**	**Acc**	**AUC**	**Acc**	**AUC**	**Acc**	**AUC**
K-means	88.51	89.63	81.61	82.40	**90.80**	94.41
Class K-means	87.36	90.79	86.21	84.13	89.66	93.40
Mean shift	89.66	92.51	85.06	**86.50**	90.80	93.34
Class mean shift	88.51	92.83	85.06	84.58	89.66	93.87
Disc mode seek	89.66	92.64	85.06	85.34	89.66	94.03
**Proposed**	**91.95**	**93.40**	**87.35**	86.27	**90.80**	**94.48**

*Bold values indicate best performance*.

### 5.6. Main Results on the Adult Dataset

Following the experimental settings of the complete child dataset, we also evaluate the proposed method and baselines on the adult dataset, with the results reported in Table 3. One could again observe that our method outperforms all comparing baselines with a sizable margin.

**Table 3:** Results on adult dataset.

**Method**	**Acc**	**AUC**
K-means (Liu et al., 2016)	72.31	71.51
Class K-means	73.85	66.48
Mean shift	72.31	68.97
Class mean shift	73.85	72.77
Disc mode seek	**75.39**	73.37
**Proposed**	**75.39**	**75.06**

*Bold values indicate best performance*.

### 5.7. ROC Curves

**Figure 4:**
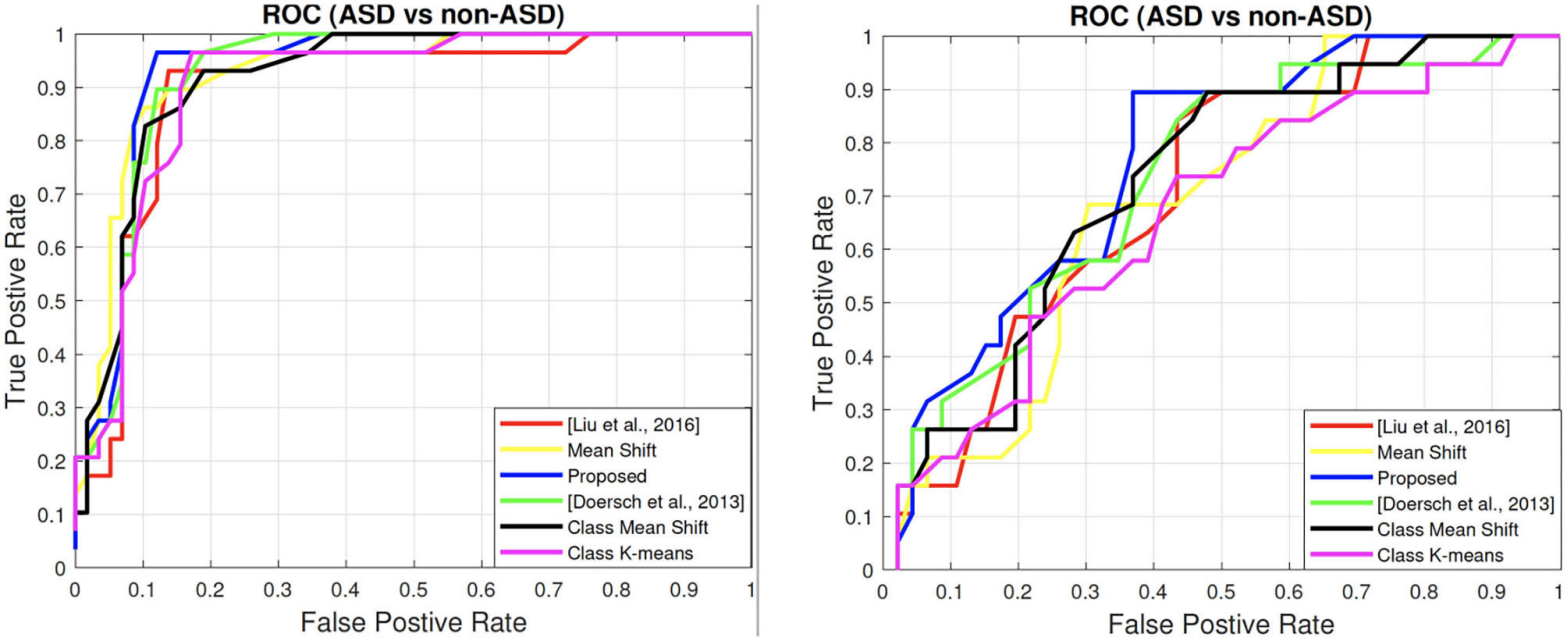
ROC Curves of all comparing methods. **(Left)** Child dataset. **(Right)** Adult dataset. Best viewed in color.

We show the ROC curves of all the comparing methods on both the child dataset and the adult dataset in Figure 4. In general, an ROC curve closer to top left corner indicates the better prediction quality of a model. This can be quantified by the AUC score, an better reflection of the holistic ROC curve performance than accuracy since AUC is a cumulative measure over the entire range of thresholds. Overall, one could see that our method (in blue color) gives the best performance in the ASD and non-ASD classification task. The corresponding AUC scores are shown in both Tables 2, 3. The results show that the AUC scores are 93.4% on the child dataset and 75.06% on the adult dataset.

### 5.8. Sensitivity and Specificity

We report the sensitivity and specificity scores of all comparing methods in Table 4, where the proposed method overall outperforms comparing methods on both child data with *Sensitivity* = 0.966 and on adult data *Sensitivity* = 0.316. The high sensitivity means that our proposed method have few false negative results, and thus fewer cases of disease are missed. The sensitivity is very important for effective screening program. And result shows that proposed machine learning method would be useful for ASD early screening. We also discuss the performance difference on child and adult dataset on section 5.12.

**Table 4:** Sensitivity and specificity scores on child dataset and adult dataset.

**Dataset**	**Child dataset**		**Adult dataset**	
**Eval metric**	**Sensitivity**	**Specificity**	**Sensitivity**	**Specificity**
K-means (Liu et al., 2016)	0.931	0.862	0.158	0.957
Class K-means	0.966	0.897	0.158	**0.978**
Mean shift	0.862	**0.914**	0.211	0.934
Class mean shift	0.828	**0.914**	0.263	0.934
Disc mode seek	0.897	0.897	0.263	0.957
**Proposed**	**0.966**	0.897	**0.316**	0.934

*Bold values indicate best performance*.

### 5.9. Sensitivity to SVM Parameters

Although slightly different optimal configurations may apply for different methods, we observe a general trend that all comparing methods tend to work best around γ = 2^−3^ ~ 2^−4^ and *C* = 2^13^ ~ 2^14^. We also observe a clear pattern for every method that similar top results appear with multiple combinations of γ − *C* pairs: increasing γ requires decreased *C*. Most importantly, all comparing methods are not sensitive to the parameters—usually with a universal 1 ~ 2% decrease of performance within a large parameter range.

### 5.10. Dictionary Purity Analysis

**Figure 5:**
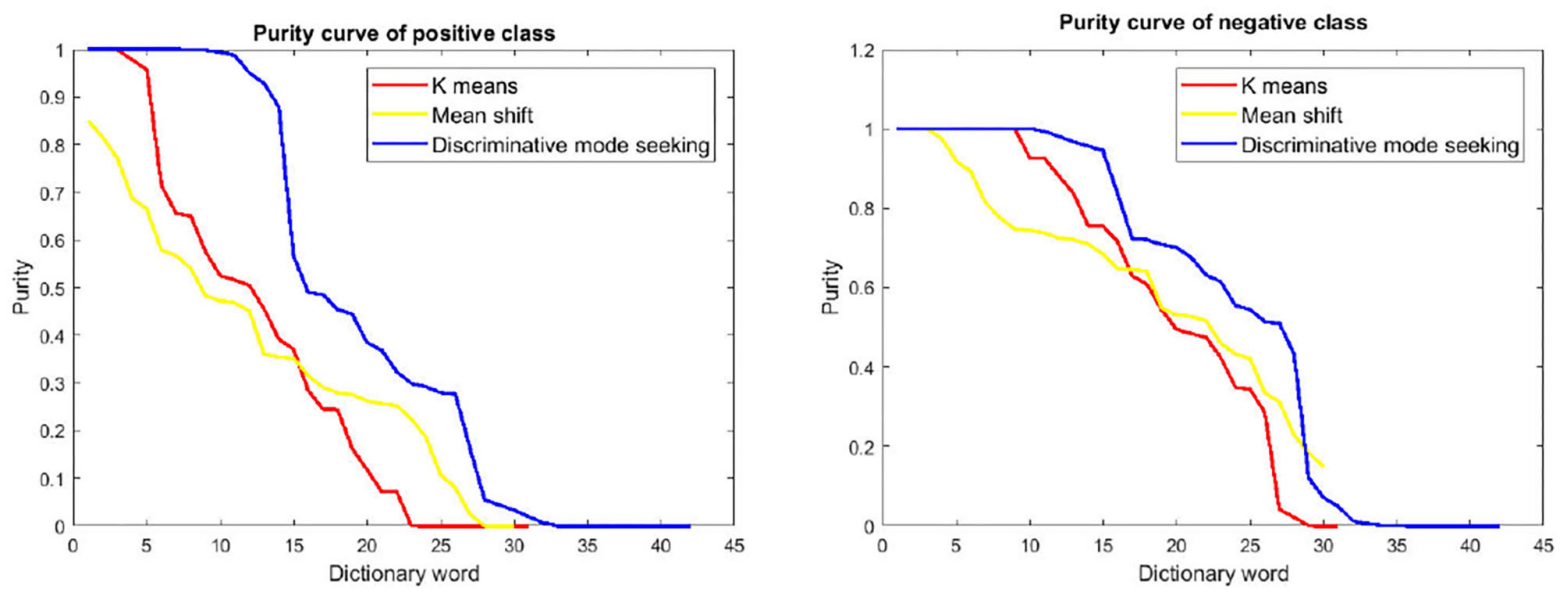
Positive and negative purity of different dictionary learning methods on child dataset. **(Left)** Purity curves of positive class. **(Right)** Purity curves of the negative class. Best viewed in color.

To analyze the discriminativeness of the dictionaries learned by different methods, we also compare the word purities of different methods in Figure 5. In particular, we first sort the dictionary words from high to low by the positive class purity, and then plot the purity of the top ranked words. One could see from Figure 5 that the proposed discriminative mode seeking method tends to have higher purities on than the others. This shows a clear evidence that the proposed method is able to explore the desterminative during dictionary learning.

### 5.11. Mode Seeking Visualization

**Figure 6:**
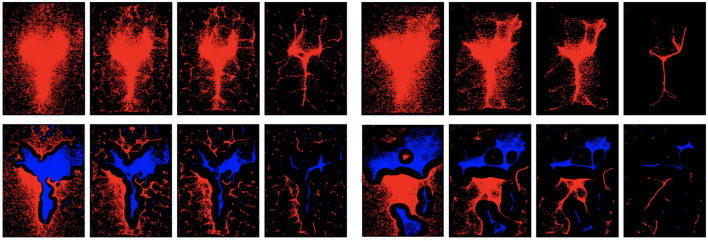
Visualization of traditional mean shift (top) and the proposed dual mode seeking (bottom) at different iterations. For dual mode seeking, red indicates $\hat{p}\left(x_{i}|X^{+}\right)>\hat{p}\left(x_{i}|X^{-}\right)>0$, while blue indicates $\hat{p}\left(x_{i}|X^{+}\right)<\hat{p}\left(x_{i}|X^{-}\right)$. **(Left)** Visualization on child dataset. **(Right)** Visualization on adult dataset. Every set of four images correspond to the visualization of shifted samples at iteration 1, 5, 10, and 30 in mean shift or the proposed method. Best viewed in color.

To show how the proposed dual mode seeking works, we visualize the shifted samples at different iterations and compare with mean shift in Figure 6. For the results of dual mode seeking, samples with red color indicates that their initial location before shifting belongs to the positive domain, while samples with blue color indicates the opposite. One could see that dual mode seeking is able to correctly find both the positive modes and the negative modes belonging to different classes. However, mean shift tends to find regions with densest samples without considering discriminative class information. It is also very interesting to see that on child Dataset, samples with higher density of negative class tend to concentrate near eyes and the center of the face, which again verifies the strong tendency of less direct eye contacts with ASD children.

### 5.12. Performance of Child Dataset vs. Adult Dataset

Upon comparing the overall identification accuracies on child dataset (Table 1) and adult dataset (Table 3), one could observe that the performance on adult dataset is not as good as the performance on child dataset. We suspect that when viewing face images, children's reactions are generally more spontaneous than those of adults. Adults having ASD may have experienced years of clinical intervention and social training. Such intervention can be a likely cause that makes the eye gaze patterns of adults less discriminative from typically-developed ones. This suspect could also be verified by comparing the visualization of dual mode seeking on both child dataset and adult dataset. One could find discriminative regions on the result of child dataset, while the result on adult dataset tends to have less discriminative regions.

## 6. Discussions and Remarks

The Experimental results indicate that our model gives considerable improvement over several widely used dictionary learning methods in terms of representing the face scanning patterns for ASD identification. On the child dataset, our method achieves an accuracy of 91.95% and an AUC score of 93.4%. On different subsets of the child dataset (different TD groups and different face race subsets), our method also outperforms different baselines. The sensitivity and specificity scores of different methods show that our proposed method has the highest sensitivity which may benefit early ASD screening since fewer cases of positive will be missed. However, we notice that the performance on the adult dataset is less promising compared to the child dataset. The conjecture of such observation is stated in section 5.12.

When comparing among the baselines, one could observe a general trend that the methods based on mode seeking (mean shift, class mean shift, discriminative mode seeking, and the proposed method) tend to outperform k-means based method since they generate arbitrary shaped dictionary clusters that better capture important patterns in the feature space. On the other hand, methods based on k-means assume more regular shaped dictionary clusters which are less discriminative. In addition, the connection between mode seeking based methods and the iMap approach (Caldara and Miellet, 2011) also partly explains the popularity of iMap in the behavioral research community from a pattern recognition perspective.

## 7. Conclusions

In this paper, we propose a novel dictionary learning method based on discriminative mode seeking. Our method incorporates label information and can automatically mine discriminative dictionary words through supervised mean shift. We also give detailed motivation, intuition, as well as links to psychology studies for the proposed method. Our method can be extended to other types of features as well. For example we could apply the same dictionary learning and BoW representations to motion features and short coordinate sequences in order to incorporate short temporal and higher order information. In addition, the datasets used in this work only contain with children between age 5 and 10 and adults, with the races of the viewed faces limited to Asian and Caucasian. Including participants with a wider range of ages (especially children), races and genders, together with designing a more comprehensive test protocol, will help to better mitigate the dataset biases and consolidate the psychological discoveries. We will leave this to be addressed and studied in future work.

## Data Availability Statement

Publicly available datasets were analyzed in this study. The data was provided by Yi et al. (2015).

## Ethics Statement

The studies involving human participants were reviewed and approved by DKU IRB. Publicly available datasets (cleaned eye tracking coordinates only with ASD labels, non-identifiable) provided by Yi et al. (2015) were analyzed in this study.

## Author Contributions

WL contributed to the design machine learning method and implementation of the research, and to the writing of the manuscript. ML and BR involved in planning and supervised the work and contributed to the writing of the manuscript. XZ contributed to the ASD protocol design. All authors contributed to the article and approved the submitted version.

## Funding

This research was funded in part by the National Natural Science Foundation of China (62171207, 61773413), Science and Technology Program of Guangzhou City (201903010040, 202007030011), the Fundamental Research Funds for the Central Universities (2042021kf0039), Key Research and Development Program of Jiangsu Province (BE2019054), and Six Talent Peaks Project in Jiangsu Province (JY-074).

## Conflict of Interest

The authors declare that the research was conducted in the absence of any commercial or financial relationships that could be construed as a potential conflict of interest.

## Publisher's Note

All claims expressed in this article are solely those of the authors and do not necessarily represent those of their affiliated organizations, or those of the publisher, the editors and the reviewers. Any product that may be evaluated in this article, or claim that may be made by its manufacturer, is not guaranteed or endorsed by the publisher.

## Footnotes

^1^Our input are x-y coordinates with the dimension of 2.

^2^One may also consider linear combination but this leads to the weight issue between purity and coverage for the different scales.
